# Case report: Novel FHR2 variants in atypical Hemolytic Uremic Syndrome: A case study of a translational medicine approach in renal transplantation

**DOI:** 10.3389/fimmu.2022.1008294

**Published:** 2022-11-14

**Authors:** Emma Diletta Stea, Christine Skerka, Matteo Accetturo, Francesco Pesce, Thorsten Wiech, Andrea Hartman, Paola Pontrelli, Francesca Conserva, Giuseppe Castellano, Peter F. Zipfel, Loreto Gesualdo

**Affiliations:** ^1^ Department of Emergency and Organ Transplantation, Nephrology and Urology Units, University of Bari Aldo Moro, Bari, Italy; ^2^ Department of Infection Biology, Leibniz Institute for Natural Product Research and Infection Biology, Jena, Germany; ^3^ Section of Nephropathology, Institute of Pathology, University Hospital Hamburg-Eppendorf, Hamburg, Germany; ^4^ Institute of Microbiology, Friedrich-Schiller-University, Jena, Germany

**Keywords:** complement associated kidney diseases, complement inhibition, Factor H related proteins, thrombotic microangiopathy, aHUS

## Abstract

Atypical hemolytic–uremic syndrome (aHUS) is a severe thrombotic microangiopathy in which kidney involvement is common. aHUS can be due to either genetic or acquired abnormalities, with most abnormalities affecting the alternative complement pathway. Several genetic factors/alterations can drive the clinical presentation, therapeutic response, and risk of recurrence, especially recurrence following kidney transplantation. We report here the case of a 22-year-old man who developed a severe form of aHUS. Renal biopsy revealed thrombotic microangiopathy and features of chronic renal damage. Despite two eculizumab infusions, the patient remained dialysis dependent. Two novel rare variants, c.109G>A (p.E37K) and c.159 C>A (p.Y53*), were identified in the factor H-related 2 (*FHR2*) gene, and western blot analysis revealed a significant reduction in the level of FHR2 protein in the patient’s serum. Although FHR2 involvement in complement 3 glomerulopathy has been reported previously, a role for FRH2 as a complement modulator has not yet been definitively shown. In addition, no cases of aHUS in individuals with *FHR2* variants have been reported. Given the role of FHRs in the complement system and the fact that this patient was a candidate for a kidney transplant, we studied the relevance of low FHR2 plasma levels through a set of functional *in vitro* assays. The aim of our work was to determine if low FHR2 plasma levels could influence complement control at the endothelial surface with a view to identifying a therapeutic approach tailored to this specific patient. Interestingly, we observed that low FHR2 levels in the patient’s serum could induce complement activation, as well as C5b–9 deposition on human endothelial cells, and affected cell morphology. As C5b–9 deposition is a prerequisite for endothelial cell damage, these results suggest that extremely low FHR2 plasma levels increase the risk of aHUS. Given their ability to reduce C5b–9 deposition, recombinant FHR2 and eculizumab were tested *in vitro* and found to inhibit hemolysis and endothelial cell surface damage. Both molecules showed effective and comparable profiles. Based on these results, the patient underwent a kidney transplant, and received eculizumab as induction and maintenance therapy. Five years after transplantation, the patient remains in good general health, with stable graft function and no evidence of disease recurrence. To our knowledge, this is first reported case of an aHUS patient carrying *FHR2* mutations and provides an example of a translational therapeutic approach in kidney transplantation.

## Introduction

Atypical hemolytic–uremic syndrome (aHUS) is a systemic endotheliosis that is characterized by severe microangiopathy and renal failure, leading to uncontrolled activation of the alternative complement pathway (1, 2). The causes of this severe kidney disease are genetic abnormalities, mostly of complement genes, and acquired factors (autoantibodies), most of which affect the binding affinity of the central regulator, factor H, to the cellular surface. The imbalance between complement activators and regulators leads to enhanced lytic complement complex (C5b–9) deposition in the vasculature, primarily in glomerular blood vessels. Vascular damage drives kidney dysfunction (1–3) and, therefore, blockade of distal complement and of lytic C5b–9 is the major goal of current therapeutic approaches, for example with eculizumab or ravulizumab.

The specific or individual genetic background of patients with aHUS influences the subtle effects of complement action, thus affecting therapeutic responses, recurrence rates, and prognosis throughout the entire process of renal transplantation (4, 5). In transplant settings, some environmental triggers, such as ischemia–reperfusion injury, acute rejection, and immunosuppressive drugs, induce activation of the complement system and contribute to disease recurrence and graft loss (6).

The risk of aHUS recurrence on the allograft ranges from less than 20% to more than 90% depending on the patient’s genetic background and the therapeutic strategy. Recipients with mutations in the genes encoding circulating complement factors [i.e., C3 (C3), factor H, factor I, factor B] have a higher risk of recurrence than patients with genetic variants encoding membrane and local modulators of the complement system, such as cluster of differentiation 46 (CD46) (6–8).

Zuber et al. (8, 9) suggested that patients with aHUS can be divided into three different categories according to the risk of post-transplant disease recurrence. Mutations in factor H, rearrangement in factor H/factor H-related (*FHR*) genes, gain of function mutations in C3 and complement factor B, and a previous history of aHUS recurrence were found to be associated with a high recurrence risk, and this was confirmed Kidney Disease Improving Global Outcomes (KDIGO) working group, which recommends the prophylactic use of eculizumab on the day of transplantation to prevent overactivation of the complement system (10). In these puzzle-like scenarios, the discovery of novel genes and of novel risk variants associated to aHUS is of high interest and significance. To determine the most appropriate therapeutic interventions in an individual patient, especially in a transplant setting, investigation of the exact genetic background, gene risk haplotypes, and autoimmune factors is extremely helpful, as the prophylactic use of eculizumab, ravulizumab, or plasma therapy remains at clinicians’ discretion (10).

Here, we present the first case of a patient with aHUS carrying two novel *FHR2* gene variants and with severely reduced plasma levels of FHR2. Eberhardt et al. (11–13) have shown that FHR2, a novel complement modulator and inhibitor, inhibits C3 convertase and blocks C5b–9 assembly. However, the exact role of FHR2 in complement modulation, in particular how it interacts with other FHRs (i.e., multimer formation with FHR1 and FHR5) and with complement components, such as C3, the cleavage products C3b and C3d, and its involvement in aHUS pathogenesis are still unclear.

Faced with a patient who was a candidate for a kidney transplant, we performed a set of functional assays *in vitro* to determine if extremely low FHR2 plasma levels can influence complement activation and C5b–9 deposition on endothelial cellular surfaces, and, if so, whether or not these effects could be reversed by restoring FHR2 levels or by eculizumab. We then used the results of the *in vitro* assays to inform and formulate the best therapeutic approach for this specific patient. The *in vitro* tests showed that extremely low plasma FHR2 levels can induce complement activation and enhance C5b–9 deposition on endothelial cells, suggesting that low FHR2 levels can form the basis for aHUS onset. Moreover, both recombinant FHR2 and eculizumab were able to control complement action in the patient’s plasma, and to reduce endothelial C5b–9 deposition to a similar degree. Collectively, these findings allowed us to tailor induction therapy and maintenance therapy following kidney transplant. This translational approach, based on genetic testing, circulating complement profiling, and functional *in vitro* experiments, provides a novel framework that is useful to optimize the targeted treatment of complement dysfunction in the clinical setting.

## Case report

A 24-year‐old Caucasian man was referred to the nephrology unit for a pre-transplantation kidney evaluation. At the age of 22 years, the patient had been admitted to hospital following the sudden development of swelling of the face and legs, epistaxis, postural instability, chest pain, purpuric rush on both arms, and high blood pressure (180/90 mmHg). The patient reported flu-like symptoms without fever during the previous 2 weeks. Laboratory analysis revealed hemolytic anemia (i.e., hemoglobin 7.2 g/dl; schistocytes 10%, lactate dehydrogenase 1,724 U/l, haptoglobin < 0.08 g/l), thrombocytopenia (100 × 10^3^/μl), severe kidney failure (creatinine 16.85 mg/dl), and low C3 plasma levels (0.5 g/l; normal range = 0.8–1.8 g/l) (**Supplementary Table 1**). ADAMTS13 (a disintegrin and metalloprotease with thrombospondin type 1 motifs 13) activity was more than 5% and the verocytotoxin-producing *Escherichia coli* test was negative. Kidney measurements on ultrasound were close to the lower limits of the reference values (i.e., right kidney longitudinal axis: 10.5 cm; left kidney length: 11 cm), with a normal cortical thickness. Of note was that the patient had no family history of thrombotic microangiopathy (TMA) or other kidney diseases, and reported good health. Laboratory analyses carried out before the onset of aHUS, when the patient was aged 6 years, showed normal kidney function and no urinary abnormalities.

Kidney biopsy revealed glomerular sclerosis, diffuse interstitial fibrosis (90%), tubular atrophy, and the vascular lesions typically associated with TMA. aHUS was diagnosed. Despite blood transfusions, hemodialysis, steroid treatment, and the administration of two doses of eculizumab (900 mg/week × 2 weeks) starting 1 month after hospitalization, the patient rapidly progressed to end-stage renal disease. Given the findings of kidney biopsy, eculizumab administration was discontinued and the patient remained dialysis dependent.

## Genetics, western blot analysis, and complement assays

**Figure 1:**
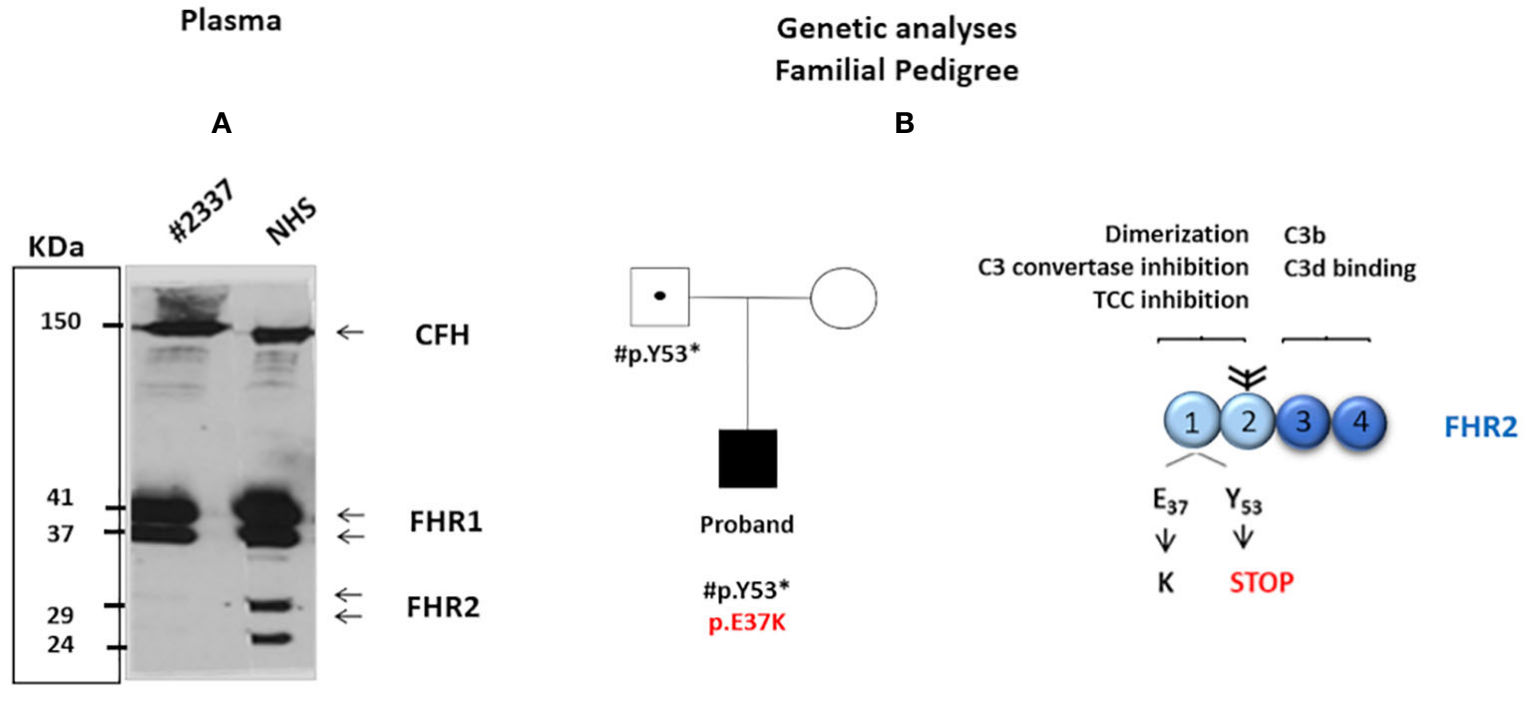
**(A)** Western blot analysis was carried out under non-reducing conditions with polyclonal anti-complement factor H-related protein 1 (FHR1). The intensity of FHR2 was much lower (around 5%) in the patient’s serum than in NHS = 100%. The positions of the two FHR2 isoform are indicated. In contrast, the intensity of complement factor H and of FHR1 appears similar in both the patient’s serum and NHS. **(B)** The patient’s family pedigree. The proband (filled symbol) carries two rare novel variants in the SCR1 of FHR2: a *de novo*p.E37K missense variant (shown in red) and p.Y53* (inherited from the patient’s father). The father is a healthy carrier of p.Y53* (dotted symbol). The patient’s mother presents no variants in FHR2 (open symbol). The structure of the FHR2 protein is shown. FHR2 is composed of four SCRs. SCRs 1 and 2 (light blue) are involved in dimer formation, C3 convertase inhibition, and terminal complement component blocking. In contrast, SCRs 3 and 4 (dark blue) bind C3b and the C3d. Two FHR2 molecules form a dimer through their N-terminal SCRs. Attached carbohydrates are indicated in black.

The screening of complement genes associated with aHUS was performed with next-generation sequencing using an in-house genetic panel, and confirmed by Sanger sequencing (see **Supplementary Material**). Significantly, the patient showed two novel variants in the *FHR2* gene: 1) a novel missense variant, c.109G>A, resulting in the exchange of E37 to K37; and, 2) a nonsense variant, c.159 C>A, which introduces a premature stop codon at position 53 (**Figure 1B**). Both c.109G>A and c.159 C>A affect the short consensus repeat domain (SCR) 1; neither is present in the Exome Variation Data Bank and neither was detected in a cohort of 60 healthy control subjects. No hybrid *FHR* genes or genomic deletions were detected by multiplex ligation-dependent probe amplification (MLPA) **Supplementary Material**.

Family studies revealed that the c.159 C>A variant introducing the stop codon was inherited from the patient’s healthy father (the patient’s healthy mother showed wild-type *FHR2*) and that the p.E37K exchange was a *de novo* variant (**Figure 1B**). These genetic variations affect FHR2 plasma levels. Western blot analysis revealed the absence of FHR2 in the patient’s serum, in contrast to a normal human serum (NHS), and normal intensity and mobility of factor H (**Figure 1A**). On longer blot exposure, however, the two FHR2 bands could be identified in the patient’s sample. Densitometry scan analysis revealed that FHR2 levels in the patient’s plasma were about 5% of those in NHS (**Figure 1A**). FHR1, FHR3, FHR4, and FHR5 levels were comparable to those of NHS (data not shown).

## Functional *in vitro* tests

Considering the possibility of a kidney transplant, we then performed a set of functional assays *in vitro* to investigate the impact of FHR2 variations on catabolite activator protein Complement Alternative Pathway (CAP) modulation and to define the specific therapeutic approach for our patient.

**Figure 2:**
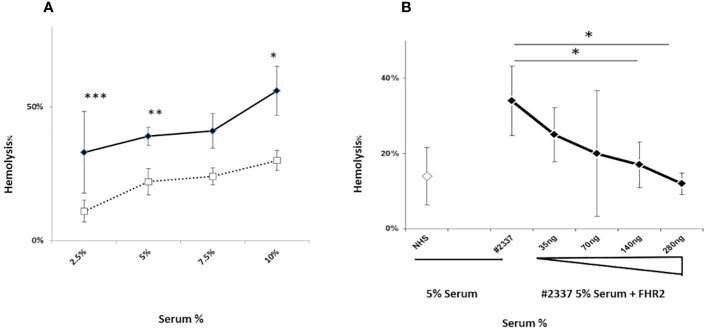
A FHR2 defect results in activation the complement alternative pathway. **(A)** Lysis of guinea pig erythrocytes. Guinea pig erythrocytes were incubated with increasing concentrations (2.5%, 5%, 7.5%, or 10%) of patient serum HR#2337 (filled rhombus) or NHS (open square). Lysis was determined by measuring the absorbance of released hemoglobin at 414 nm. The patient’s serum induced more erythrocyte lysis (33%, 39%, 41%, and 56%) than NHS (11%, 22%, 24%, and 30%). The difference was statistically significant at serum concentrations of 2.5%, 5%, and 10%. The results are mean ± standard deviation (SD) of three independent experiments. *p < 0.05, **p < 0.01, ***p < 0.001. **(B)** Guinea pig erythrocytes were incubated with 5% NHS (open rhombus) or 5% HR#2337 serum (filled rhombus) alone or enriched with increasing amounts (35 ng, 70 ng, 140 ng, or 280 ng) of recombinant FHR2. The addition of recombinant FHR2 reduced erythrocyte lysis from 34% to 12%. The results are mean ± SD of three independent experiments. *p < 0.05.

High levels of the complement activation fragments C3a, Ba, and sC5b–9 were detected in the patient’s serum, suggesting CAP activation (**Supplementary Material** and **Supplementary Figures 1A–C**). To investigate this further, we used a guinea pig model to measure the degree of hemolysis induced by adding different concentrations (i.e. 2.5%, 5%, 7.5%, and 10%) of fresh patient serum (HS#2337) or NHS to guinea pig blood *in vitro* (methods described in **Supplementary Material**). The patient’s serum induced more erythrocyte lysis (33%, 39%, 41%, and 56%) than NHS (11%, 22%, 24%, and 30%), confirming CAP activation. The effect was dose dependent, and differences at serum concentrations of 2.5%, 5%, and 10% were statistically significant (**Figure 2A**).

Next we investigated the role of a low plasma level of FHR2 in the modulation of the alternative pathway, testing whether the addition of FHR2 to active HS#2337 restores the hemolytic effect. The hemolytic assay was repeated, incubating 5% patient serum with increasing amounts of recombinant FHR2 (from 0 to 280 ng). The negative control was 5% NHS. When recombinant FHR2 was added to the HS#2337, erythrocytes lysis was reduced from 34% to 12%, suggesting a role for FHR2 as a surface regulator of the alternative pathway (**Figure 2B**). The effect was dose dependent, and the lytic activity of the patient’s serum was fully restored by the addition of 140–280 ng of FHR2. As the systemic endotheliosis induced by a high level of C5b–9 cell deposition represents a milestone in the pathogenesis of aHUS, we evaluated C5b–9 deposition using human umbilical vein endothelial cells (HUVECs) incubated with patient serum and with NHS, using confocal microscopy and flow cytometry (methods described in **Supplementary Material**).

**Figure 3:**
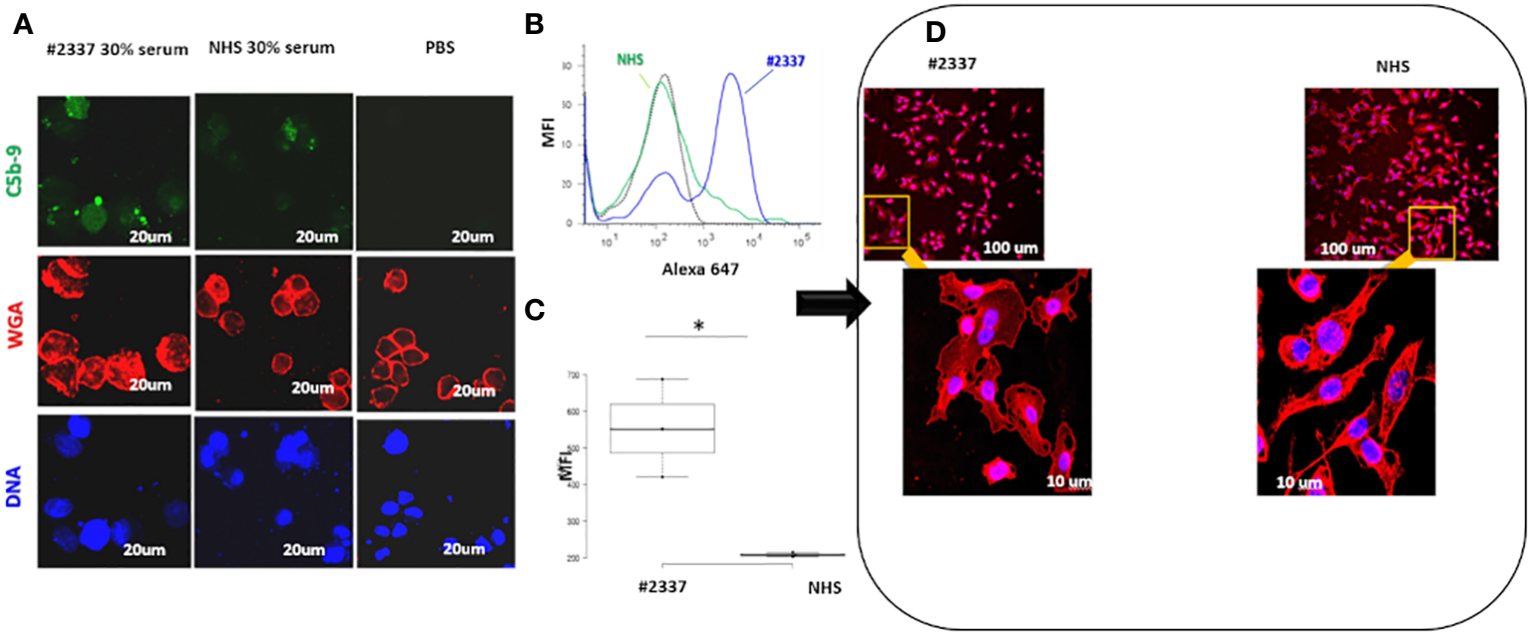
Low serum FHR2 levels result in enhanced C5b–9 deposition on HUVECs, modifying cell morphology. **(A)** HUVECs incubated with 30% HS#2337 (Laser Scanning Microscopy, LSM) showed greater deposition of C5b–9 (green fluorescence) than cells that were incubated with 30% NHS. Nuclei were stained with 4′,6-diamidino-2-phenylindole (DAPI) (blue fluorescence). The intact membrane was stained with wheat germ agglutinin Texas Red (WGA) (red fluorescence). Scale bar, 20um. A result representative of three independent experiments is shown. **(B)** Fluorescence-activated cell sorting (FACS) confirmed the results obtained with LSM. HUVECs incubated with 30% patient serum lacking FHR2 showed greater C5b–9 deposition than HUVECs incubated with NHS. MFI, mean fluorescence intensity. **(C)** The boxplot displays the Mean Fluorescence Intensity (MFI) of HUVEC incubated with either the patient's serum (white) or NHS serum (grey). Data for the triplicate experiments is shown. *p < 0.05. **(D)** HUVECs were incubated with HS#2337 or NHS. Cell morphology was determined by examination of WGA fluorescence using Laser scanning microscopy. Cells exposed to HS#2337 are retracted, losing their typical extended shape, and have more intracellular inclusions. Scale bar 100 μm and 10 μm.

As shown in **Figure 3A**, C5b–9 fluorescence was intense and diffuse on endothelial cells exposed to HS#2337, was minimal on cells incubated with NHS, and was absent on cells incubated with phosphate-buffered saline. Moreover, HUVECs incubated with 30% HS#2337 presented a loss of the typical shape, and their usual morphology was lost (**Figure 3D**).

**Figure 4:**
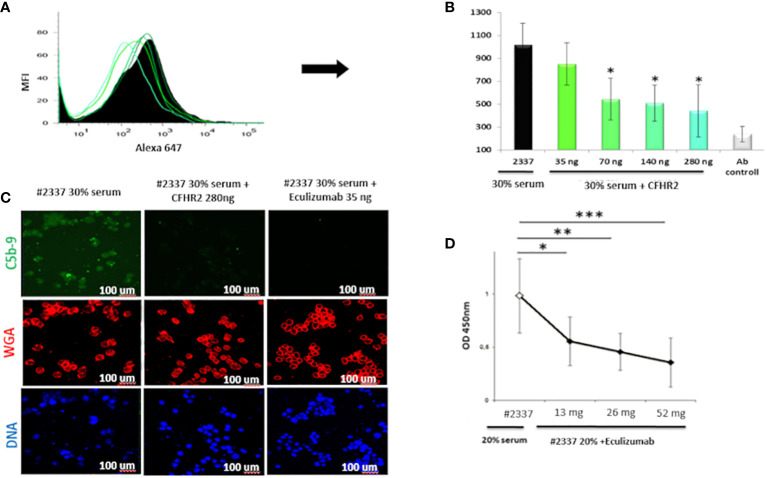
Recombinant FHR2 and eculizumab block C5b–9 deposition by HS#2337. **(A, B)** Endothelial cells were incubated with HS#2337 (30%) for 30 minutes. An increasing amount of recombinant FHR2 was added to HS#2337 serum and C5b–9 signaling was examined by flow cytometry. Antibody (Ab) control represents no interaction. The recombinant FHR2 reduced, in a dose-dependent manner, C5b–9 deposition on HUVECs, and this reduction was statistically significant at 70 ng, 140 ng, and 280 ng of FHR2. MFI, mean fluorescence intensity. The results are mean ± standard deviation (SD) of three independent experiments. **(C)** The effects of eculizumab and FHR2 on C5b–9 deposition was compared by laser scanning microscopy. HUVECs were incubated with 30% HS#2337serum, with 30% HS#2337serum premixed with 280 ng of FHR2 and with 30% HS#2337 serum premixed with eculizumab (35 ng). C5b–9 deposition was evaluated using the monoclonal C5b–9 antibody. **(D)** Increasing amounts of eculizumab (from 0 to 52ng) were added to 20% HS#2337, and the effect on C5b–9 generation was evaluated by ELISA. Eculizumab significantly reduced C5b–9 generation. The effect is dose dependent. Results represent mean ± SD of three independent experiments (see **Supplementary Material** for more details). Eculizumab and FHR2 reduced C5b–9 deposition (green fluorescence) on endothelial cells to a similar degree. Nuclei were stained with 4′,6-diamidino-2-phenylindole (DAPI) (blue fluorescence). Intact membrane was stained with wheat germ agglutinin Texas Red (WGA) (red fluorescence). Scale bar, 100 μm. The result is representative of three independent experiments. *p < 0.05, **p < 0.01, ***p < 0.001.

The results were confirmed by flow cytometry. Endothelial cells exposed to 30% patient’s serum showed a C5b–9 signal 2-fold higher than that observed when incubating the cells with NHS [mean fluorescence intensity (MFI) express in percentage: HS#2337, 554; NHS, 208] (**Figures 3B, C**). The difference was statistically significant (*p* ≤ 0.05). As low FHR2 levels reduce complement regulation, causing more C5b–9 deposition, we carried out an assay to determine whether or not recombinant FHR2 was able to reverse the strong C5b–9 deposition induced by HS#2337. To this end, HUVECs were incubated with 30% patient serum to which increasing amounts of recombinant FHR2 (from 0 to 280 ng) were added, and C5b–9 signaling was evaluated by flow cytometry. *In vitro* addition of FHR2 to HS#2337 reduced HUVECs C5b-9 deposition in a dose-dependent manner. When FHR2 was used at a concentration of 280ng, C5b-9 signaling on HUVECs was completely lost (MFI: HS#2337, 1017; HS#2337 + 280 ng FHR2, 442) (**Figures 4A, B**). Thus, adding FHR2 to the patient’s serum restored the complement modulation *in vitro*, re-establishing the physiological state.

Considering the poor response to eculizumab during aHUS onset, we also assayed whether or not the C5-blocking complement inhibitor can also effectively reduce the high C5b–9 levels observed in the patient’s serum. Therefore, an increasing amount of eculizumab (increasing from 0 to 52 ng) was added *in vitro* to 20% HS#2337. C5b–9 levels were then measured by ELISA. *In vitro*, the drug blocked the C5b–9 generation in the plasma of our patient in a dose-dependent manner, confirming its efficacy in this unique scenario (**Figure 4D**).

Finally, we compared FHR2 and eculizumab efficacy in regulating HS#2337-induced C5b–9 deposition. HUVECs were incubated with HS#2337, with HS#2337 plus FHR2 (280 ng), and with HS#2337 plus eculizumab (35 ng). C5b–9 signaling was then evaluated by confocal microscopy (**Figure 4C**). Interestingly, eculizumab and FHR2 reduced C5b–9 deposition on endothelial cells in a similar manner (**Figure 4C**), thus offering potential future therapeutic opportunities.

## Personalized treatment strategy and patient outcome

Following 23 months of hemodialysis, the patient underwent a kidney transplant at the University Hospital of Bari (Italy), receiving a cadaveric graft from a standard criteria donor. Two (A11, B18) of six antigens were mismatched, four (A24, B35, DR11, DR14) of six human leucocyte antigens were matched, no donor-specific antibodies were detected, and the panel-reactive antibody positivity was 0% (**Supplementary Table 1**).

Based on the patient’s clinical history and genetic background, and the results of the *in vitro* assays, we defined a tailor-made therapeutic strategy. Before surgery, the patient received a single plasma exchange, thymoglobulin infusion, steroids, and one dose of eculizumab (900 mg) to confer a high level of graft protection. During the post-transplant phase, steroids were gradually reduced, and plasma exchange was replaced by plasma infusion, which was administered as thymoglobulin for 6 days. This therapeutic scheme allowed the introduction of calcineurin inhibitors (i.e., tacrolimus 0.1 mg/kg) 4 days after the transplant, attenuating the endothelial damage, coupled with mycophenolate (**Supplementary Figure 2**). Complement analysis of the patient’s serum (on the 10th day post transplant) showed low levels of activation of fragments C3a, Ba, and sC5b–9, confirming the *in vivo* efficacy of this therapeutic approach (**Supplementary Figure 3**). Eculizumab was administered weekly for 3 weeks, and then every 2 weeks for 2 months. Given the early graft function, the absence of organ rejection, and the absence aHUS recurrence, the administration of eculizumab was suspended 2 months after transplantation, and the patient was closely monitored with regular follow-up visits. During the 5 years of follow-up (**Supplementary Figure 4**), no signs of hemolysis, kidney graft dysfunction, or proteinuria were detected (**Supplementary Table 2**).

## Discussion

Kidney transplantation in patients with aHUS is associated with poor outcomes due to high recurrence rates and graft loss. The genetic background has a strong influence on the natural history of the disease (6–8). The KDIGO working group recommends the prophylactic use of eculizumab in the management of patients with aHUS who receive a kidney transplant and carry pathogenic or gain-of-function gene mutations (10). However, the graft outcome in transplanted patients with aHUS carrying novel variants, especially in emerging genes, is unpredictable, thus hampering our ability to optimize immunosuppressive therapy.

Our patient developed a severe form of aHUS after an upper respiratory infection, which is hypothesized to be the disease trigger. Unfortunately, eculizumab was administered 1 month following disease onset and, thus, therapy initiation was significantly delayed. Moreover, owing to the chronicity aspect of kidney chronocity, clinicians decided to administer only two doses of eculizumab.

Unusually, the patient described in this study carries two novel rare variants of the *FHR2* gene, and these were associated with a reduction in plasma concentration of the protein. FHR2 is a dimeric plasma protein that is composed of four SCRs, with high sequence similarity to FH, FHR1, and FHR5 (14). Eberhardt et al. (11) showed that FHR2 inhibits C3 convertase and blocks C5b–9 assembly through its N-terminal domain (SCR 1–2), which is also involved in dimer formation. Despite these findings, the exact role of FHR2 in complement modulation and aHUS pathogenesis is still under investigation, presenting an open challenge for scientists and clinicians. Our patient presented a heterozygous *de novo* missense FHR2 variant, inherited from his father, which results in amino acids exchanges in SCR1 (i.e., E37 to K37) and the introduction of a premature stop codon (c.159C> G; p.Y53*) in SCR1. These variants are located within the N-terminal domain, which mediates FHR2 dimerization and inhibition of C5b–9 assembly, suggesting an important impact on protein function (11). Moreover, p.Y53*, which corresponds to p.Y35* in the protein nomenclature without signal peptide, is located in the highly conserved first N-terminal domain of FHR2, between the residues Tyr34 and Ser36, with the nearby residue Tyr39 being critical in dimer stability (15).

The macroscopic gene rearrangement of *FHR2* with FHR1 and FHR5 has been linked to C3 glomerulopathy in three familial cases because of the expression of aberrant hybrid plasma proteins able to overactivate the CAP (16–18). In our patient with aHUS, MLPA excluded the presence of copy number variations in the *FHR2* and in the *FHR*s gene family. In addition, western blot analysis did not reveal the presence of hybrid proteins, but only a level for FHR2, suggesting the possibility of a different pathogenic scenario.

The patient also exhibited overactivation of the initial and final step of the CAP pathway, shown by low C3 plasma levels and increased serum amounts of complement activation fragments Ba, C3a, and sC5b–9. The functional impact of this FHR2 defect on CAP activation was studied using both the patient’s serum and the FHR2 recombinant protein. FHR2 serum deficiency is linked to high CAP activation, and this was revealed by a higher degree of erythrocytes lysis in the patient’s sample than in NHS. Moreover, the addition of increasing amounts of recombinant FHR2 to the patient serum gradually restored the complement control, suggesting a role of FHR2 in CAP modulation, especially on the cell surface.

It is well known that complement deregulation in endothelial cells plays a crucial role in aHUS pathogenesis. Large amounts of the terminal product of the complement cascade (C5b–9) on endothelial cells cause cell death and unmask prothrombotic agents, favoring TMA development (19). In our study, the patient’s FHR2-deficient serum induced a higher level of C5b–9 deposition on HUVECs than did NHS, leading to morphological changes in the cells. The addition of CFHR2 to the serum of our patient significantly reduced C5b–9 deposition on HUVECs in a dose-dependent manner, re-establishing complement control. Indeed, FHR2 acts a complement surface regulator, possibly supporting CFH inhibitory role during complement activation.

When it became apparent that our patient would need to undergo kidney transplantation, we evaluated the opportunity to administer a C5b-blocking complement inhibitor. Thus, we investigated the effect of eculizumab on C5b–9 serum levels and deposition of C5b–9 on cells. Through a series of different *in vitro* assays, we demonstrated that eculizumab was effective in reducing C5b–9 signaling in this specific patient with FHR2 deficiency, as demonstrated through ELISAs and observation of HUVECs incubated with the patient’s serum. Interestingly, eculizumab and the recombinant FHR2 showed a comparable effect on controlling the complement system, indicating their potential benefits for the treatment of these patients.

Based on the results of these biological tests, we decided to prophylactically administer eculizumab plus plasma therapy before the transplant surgery (20). The prophylactic use of combined eculizumab plus plasma therapy has been reported in a small number of transplant patients with severe aHUS with a severe phenotype, but further studies in larger cohorts are needed to confirm its utility in preventing disease recurrence and graft loss in this group of patients (10, 21).

Plasma therapy was administered for 6 days after transplantation, whereas eculizumab was used for 2 months to confer a higher protection from complement activation. The discontinuation of eculizumab is debated, and depends on genetic variants, clinical history, and laboratory tests (22). Although two trials have recently shown the usefulness of eculizumab administration for 2 years in patients with aHUS (23), there is no clear evidence on the best timing for discontinuation, especially after kidney transplantation. The time frame for aHUS post-transplant recurrence ranges from 3 days to 6 years, with a median of 2 months. Here, we successfully discontinued eculizumab therapy 2 months after transplantation. At 5 years’ follow-up, the patient showed normal graft function and no signs of disease recurrence (**Supplementary Table 2**). Short-term preventive eculizumab therapy with plasma infusions was administered based on the results of specific laboratory tests, described above, and these tests (not available routinely) allowed us to set up a tailor-made treatment protocol in a patient carrying an unknown gene variant.

In summary, we reported the first case of aHUS in a patient with a genetic defect within FHR2 that seems associated with very low plasma level of this protein. We successfully transplanted the patient using personalized induction and maintenance therapy. Moreover, the analyses carried out in this index clinical case allowed us to investigate in depth the role of the FHR2 surface complement regulator, and to demonstrate that loss of/reduction in this factor may contribute to the pathogenesis of aHUS. This unique patient provides an example of the application of a translational medicine framework that could be useful in managing patients with aHUS carrying novel genetic variants of unknown significance, with the aim of defining the best therapeutic strategy in those undergoing kidney transplantation.

## Data availability statement

The original contributions presented in the study are included in the article/**Supplementary Material**. Further inquiries can be directed to the corresponding author.

## Ethics statement

Ethics review and approval were not required for the study on human participants in accordance with the local legislation and institutional requirements. The patients/participants provided their written informed consent to participate in this study. Written informed consent was obtained from the individual(s) for the publication of any potentially identifiable images or data included in this article.

## Author contributions

ES collected the data, created the figures, and drafted the manuscript with PFZ and CS. ES carried out the experiments with support from AH. MA, PP, FC and GC supported the genetic analysis and the collection of clinical and biological materials. FC provided linguistic and scientific support during revision. TW provided the kidney biopsy and supported the experimental project design. FP revised all statistical analyses and helped draft the manuscript. PFZ and LG supervised the project. All authors discussed the results and contributed to the final manuscript. LG, GC, and ES defined the therapeutic strategy for patient #2337 during the kidney transplantation. All authors contributed to the article and approved the submitted version.

## Funding

PFZ and TW received funding by the Collaborative Reserch Center CRC 1192, by the Deutsche Forschungsgemeinschaft. PFZ received support from KIDNEEDS Iowa, USA. ES was supported by a scholarship from the Società Italiana di Nefrologia (Rome, Italy).

## Acknowledgments

We thank the patient and his family for participating in the study and for contributing valuable specimens. We thank Monika von der Heide, Hans-Martin Dahse, and Ina Löschmann (Department of Infection Biology, Leibniz Institute for Natural Product Research and Infection Biology, Jena, Germany) for excellent technical support.

## Conflict of interest

The authors declare that the research was conducted in the absence of any commercial or financial relationships that could be construed as a potential conflict of interest.

## Publisher’s note

All claims expressed in this article are solely those of the authors and do not necessarily represent those of their affiliated organizations, or those of the publisher, the editors and the reviewers. Any product that may be evaluated in this article, or claim that may be made by its manufacturer, is not guaranteed or endorsed by the publisher.
