# Supplementary figures and images for: Case report: Novel FHR2 variants in atypical Hemolytic Uremic Syndrome: A case study of a translational medicine approach in renal transplantation
Source: Front Immunol. 2022 Nov 14;13:1008294. doi: 10.3389/fimmu.2022.1008294 (PMC9703090; doi:10.3389/fimmu.2022.1008294)

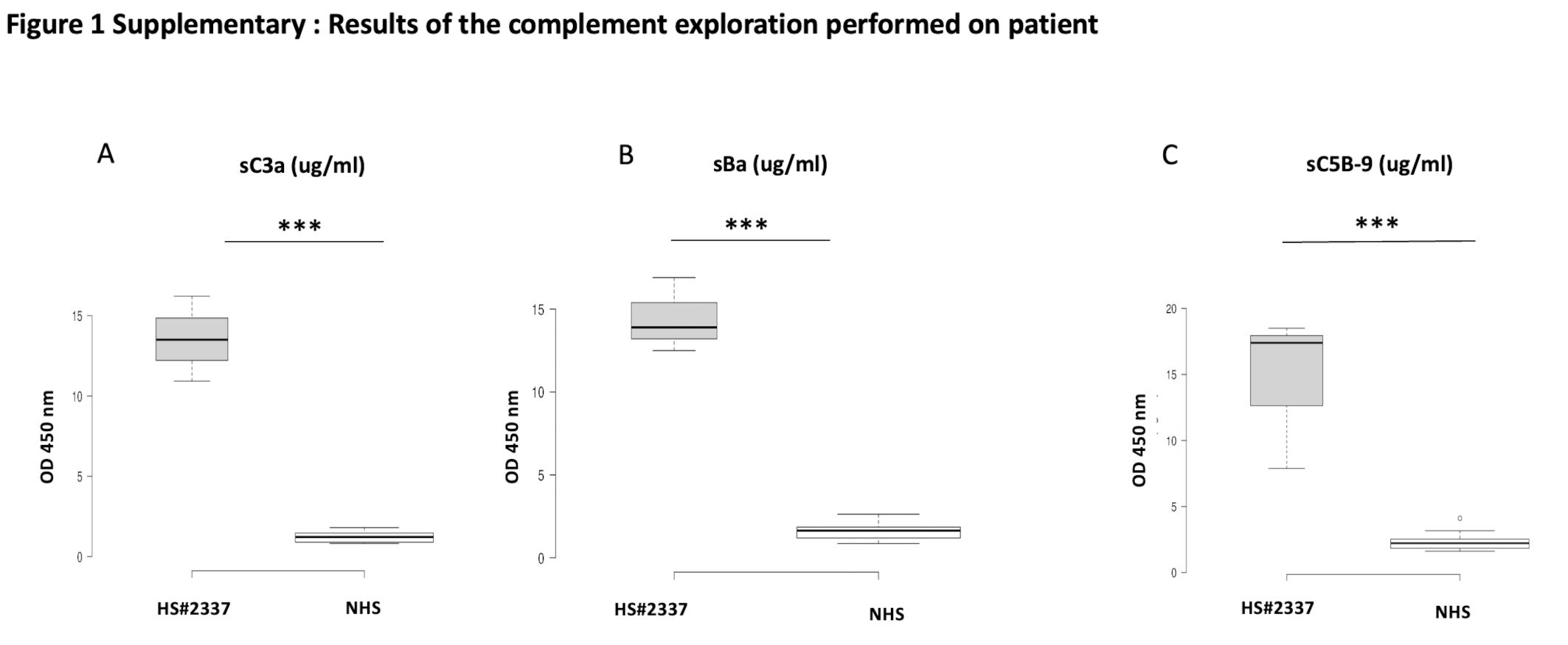

Supplement: Supplementary file 1 [file Image_1.jpeg]

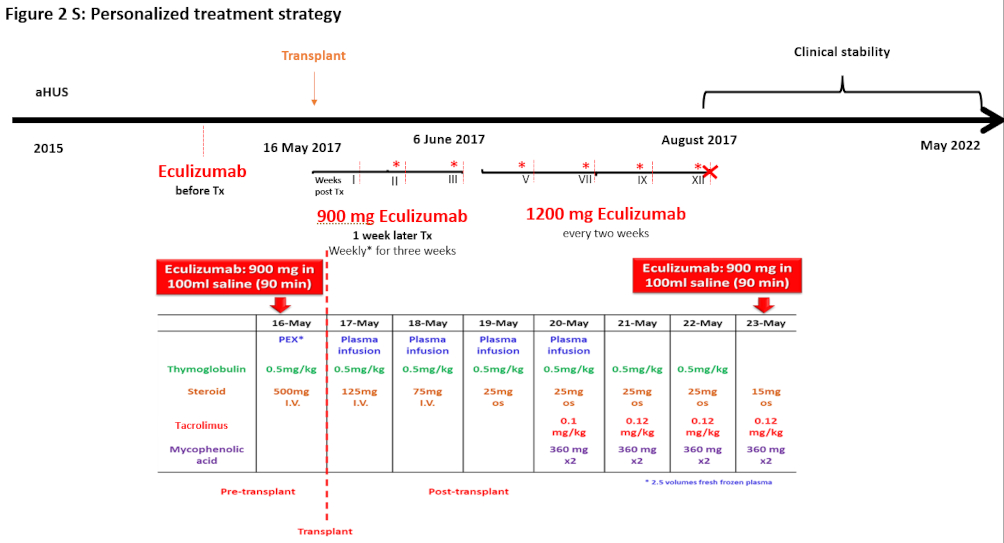

Supplement: Supplementary file 2 [file Image_2.jpeg]

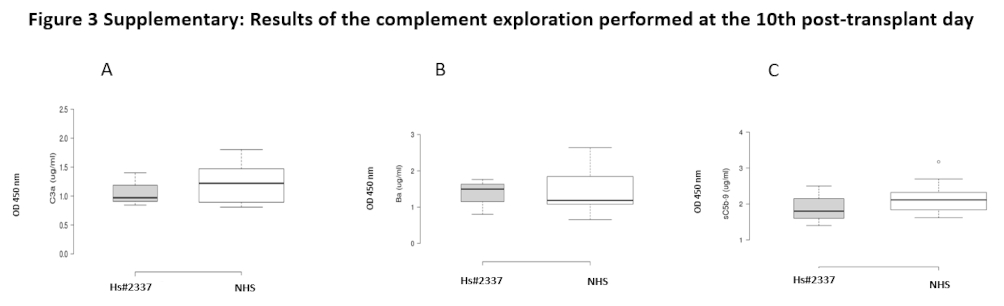

Supplement: Supplementary file 3 [file Image_3.jpeg]
