# Supplementary material for: Case report: Novel FHR2 variants in atypical Hemolytic Uremic Syndrome: A case study of a translational medicine approach in renal transplantation
Source: Front Immunol. 2022 Nov 14;13:1008294. doi: 10.3389/fimmu.2022.1008294 (PMC9703090; doi:10.3389/fimmu.2022.1008294)
Supplement: Supplementary file 4 [file DataSheet_1.docx]

***Supplementary Material***

# Supplementary Data

The paper adheres to the Declaration of Istanbul. The patient received a cadaveric graft from a standard criteria donor.

**1.1 Genetic analysis:** Patient, relatives and control DNA was extracted from peripheral blood using the PAX gene Blood DNA kit according to the manufacturer’s instructions (Quiagen). For Next Generation Sequencing (NGS): target genomic enrichment was performed using an Illumina TruSeq Custom Amplicon panel designed for C3, CFB, CFI, CFH, CD46, THBD, DGKE, FHR1-5 , ADAMTS 13 setting the amplicon length: to 250 bp ( 97% coverage across all targets). Library preparation was performed according to the manufacturer’s instructions (TruSeq Custom Amplicon library preparation guide). Sequencing was performed on an Illumina MiSeq Desktop Sequencer. FastQ files generation, alignment and variant calling was performed according to the Illumina MiSeq Reporter Custom Amplicon Workflow, using the default settings: banded Smith-Waterman algorithm for BAM files generation and Genome Analysis Toolkit (GATK) for VCF files generation. Only high-quality bases were considered. These were identified by selecting only those presenting the ‘PASS’ status of the variant call quality, meaning that all quality filters (QUAL and GQ values listed in the VCF file) were passed. Annotation of variants was performed using Illumina VariantStudio software (see Resources) and CADD algorithm^38^ (for CScores computation). Evaluation of the pathogenicity or likely pathogenicity of variants was made by setting a CScore cut-off of 10.

Molecular analysis of FHR2 gene (NM_005666) was performed by polymerase chain reaction (PCR), using homemade primers and direct sequencing. PCR reaction was run in a volume of 25 μl, with 100 ng of template DNA, 1 × reaction buffer, 0.2 mM of each nucleotide, 30 pmol of primers and 0.1 U of DNA polymerase (TaqGold, Life Technologies, Calif., USA). The PCR reaction was carried out for 35 cycles, denaturated at 95 ° C for 15 s, annealed at about 57° C for 30 s and with an extension at 72 ° C for 60 s. All PCR programs include an initial denaturation of 10 min at 95 ° C and the extension lasted 7 min in the last cycle.

The PCR products were analyzed with direct sequencing using the Big-Dye Terminator kit v3.1 (Life Technologies, Calif., USA) and ABI-PRISM 3130 Genetic Analyzer (Life Technologies, Calif., USA). **A cohort of 60 healthy control subjects have been screen for the FHR2 rare variants.**

Finally, MLPA (multiplex ligation-dependent probe analysis) was performed to determine abnormal copy numbers of factor CFH/FHRs and genes encoding FHRs 3, 1, 2 and 5 using the SALSA MLPA P236-A1 ARMD Kit (MRC-Holland). Gene-Marker software v1.85 (Soft Genetics) was used for data analysis.

**1.2 Serum analysis:** Serum proteins of the patient and health individual were separated using a non-reducing condition.10 ul serum diluted in PBS (1:20) was separated in a 12% SDS/PAGE gel at 150V for 1,30 h and transferred onto a nitrocellulose blotting membrane (Thermo Fisher scientific, Waltham, MA USA). After an overnight incubation at 4°C in 5% milk in TBST (20nM tris-HCL, 136mM naCl, 0,15% Tween-20, ph 7.4), the membrane was incubated firstly with specific anti FHR1-FR2 reacting antiserum home-made antibodies (pAb FHR1 1:2000). After three wash steps for 10 min with TPBS, the membrane was incubated with HRP-coupled secondary antibody αrabbit (1:2000) for 1 hour at RT. Finally, after another round of washing with TPBS, the detection of the proteins was performed using the chemiluminescence system (Amersham) according to the manufacturer’s information. X-ray films were used for the development.

**1.3 Complement Activation Products**

The complement activation products fragments C3a, Ba and sC5b-9 were measured in the patient's serum using Microvue kits (Quidel® Corporation, San Diego, CA) according to the manufacturer’s instructions. Normal healthy subjects (NHS) were used as negative control.

**1.4 Protein expression and purification**

Recombinant FHR2 was expressed in the Pichia system as previously described by Eberhardt H.U. et al. (11)

**1.5 Hemolytic assay**

Guinea pig erythrocytes (TCS, Bioscences, Buckingam UK) (50ul) in AP buffer, veronal buffer saline (144mmol/L NaCL, 7mmol/L MgCl_2,_ 10 mmol/L ethylene glycol tetra-acetic acid [pH 7.4]) were incubated with increasing amounts (2.5%; 5%; 7.5%; 10%) of patient serum (HS#2337) vs NHS for 30 minutes at 37°C. After centrifugation, the supernatants were read at 414nm (Tecan Safire2, Tecan, Männedorf Switzerland). Erythrocytes diluted only with AP buffer were taken as blanks (= spontaneous lysis; 0% lysis) while erythrocytes incubated with dd-water were used as 100% lysis.

In addition, a hemolytic assay with an increasing amount of recombinant FHR2 added to #2337 patient serum, was used to evaluate the inhibitor effect of FHR2 on complement activation.

Considering that in NHS the normal FHR2 concentration was assumed to be 50 mcg/ml, recombinant FHR2 was added to HS#2337, reproducing the physiological condition. Therefore, an increasing amount of FHR2 (35ng; 70ng, 140ng; 280ng) was premixed with #2337 patient serum. Then the guinea pig erythrocytes in AP buffer were incubated with 5% of #2337 patient serum alone, with 5% of # 2337 patients serum enriched with recombinant FHR2 and with 5% of NHS. After 30 minutes of incubation at 37°C, and centrifugation, the supernatants were read at 414 nm.The AP activation was evaluated by the hemolysis percentage.

**1.6 Immunofluorescence analysis of C5b-9 deposition on endothelial cells (HUVEC)**

Human Umbilical Vein Endothelial Cells (HUVEC, American Tissue Culture Collection® CRL-170™) were cultured in DMEM + medium with 10 % FCS. HUVEC grown on coverslips were incubated with 30% of HS#2337 Vs NHS for 30 minutes at 37°C, HUVEC incubated with PBS were used as negative control. After 30 minutes the cells were washed with PBS by centrifugation (2500 rpm, 5 minutes RT). Then HUVEC were treated with C5b-9 monoclonal antibody ([aE11] (ab66768) Abcam) and incubated for 45 minutes at RT. After another wash step with PBS as previously described, the cells were incubated for 30 minutes with Alexa Fluor®647-labeled (Thermo Fisher Scientific). A washing step (5 minutes at 2500 rpm) with a 500 μl PBS was repeated in order to remove the unbound antibody. DAPI (Sigma-Aldrich) and WGA-Texas Red (Thermo Fisher Scientific) were used to visualize the nucleus and the cell membrane, respectively. HUVEC incubated with PBS were used as blank. The C5b-9 deposition on HUVEC surface was evaluated by confocal microscopy (Zeiss LSM 710 confocal microscope and ZEN2009 program, Jena Germany). To test whether recombinant FHR2 and Eculizumab can revert the strong C5b-9 deposition induced by the patient serum, the experiment was repeated. HUVEC were again incubated with 30% of HS#2337, 30% of HS#2337 + FHR2 (280ng) , and with 30% of HS#2337+ Eculizumab (35ng), evaluating the C5b-9 deposition as described above.

**1.7 Immunofluorescence analysis of the endothelial cells morphology**

HUVEC, grown on coverslips, were incubated with 30% of HS#2337 vs 30% of NHS for 30 minutes, at 37°C, then fixed with 3.5% formaldehyde for 10 minutes at room temperature (RT). After washing, DAPI and WGA were added and incubated at RT for 30 minutes. The slides were analyzed by confocal microscopy.

**1.8 Flow cytometry analysis of C5b-9 deposition on HUVEC**

The surface expression of C5b-9 on HUVECs exposed to HS#2337 vs NHS was quantified by flow cytometry (BD LSR II Flow Cytometer, BD, Heidelberg Germany).

Endothelial cells in suspension were incubated for 30 minutes at 37°C with 30% of #2337 patient serum and 30% of NHS. After washing (5 minutes with PBS) cells were stained with C5b-9 monoclonal antibody, and then Alexa Fluor®647-labeled (Thermo Fisher Scientific). In every step, cells were washed for 5 minutes with 500 ul of PBS. HUVEC exposed only to Ab control were used as blanks (unspecific binding). Data were analyzed by flow cytometry (BD LSR II Flow Cytometer, BD, Heidelberg Germany) and evaluated by FlowJo.

To evaluate the inhibitor effect of FHR2 on C5b-9 deposits on HUVEC, the experiment was repeated. Hence, HUVEC were incubated with HS#2337 alone and premixed with increasing amounts of recombinant FHR2 (the same range as used for haemolytic assays) and the C5b-9 signaling was quantified using FACS.

**1.9 In vitro effect of Eculizumab on C5b-9 generation**

LPS (10 µg/ml in DPBS) was immobilized on a microtiter plate (o/n, 4°C) overnight. After washing and blocking (2% BSA in DPBS) steps, the wells were incubated with 20% of HS #2337 at at 37°C for 1 hour. The 20% HS #2337 was added to the LPS-coated wells alone and after premixing with increasing amounts of Eculizumab (13ng; 26ng; 52ng). After the incubation the ELISA plate was wash. Than the mouse monoclonal C5b-9 antibody was incubated for 1h at RT. The detection was performed using HRP- secondary antibody. OD values were measured at 450 nm (Tecan Safire2, Tecan, Männedorf Switzerland).

**1.10 Statistical analysis**

Parametric variables, expressed as mean values and standard deviation (SD), were compared using Student t test (2 tailed). Every experiment was performed in triplicate and statistical significance was set at p values <0.05 (*p<0.05 **p<0.01, ***p<0.001).

# Supplementary Figures and Tables

## Supplementary Figures

**Figure S1**: **Results of the complement exploration performed on patient: A-C)** Complement C3a **(A)**, Ba **(B)** and sC5b-9 **(C)** fragments in serum of the patient (#2337) collected during the aHUS onset (light grey boxes), compared to NHS (white boxes). The patient serum shows, an activation of initial and terminal step of CAP, with increased C3a serum levels (13,5 ug/ml), Ba serum levels (14,4 ug/ml) and sC5b-9 serum levels (14,5 ug/ml). Results represent three independent experiments. ****P*<0.001

**Figure S2 Personalized treatment strategy:** Timetable of the therapeutic strategy adopting for patient #2337 during his transplantation. During pre-transplant phase plasma exchange (PEX: 2.5 volumes of fresh frozen plasma), thymoglobulin infusion (0.5 mg/Kg), steroids (500 mg) and one dose of Eculizumab (900mg), were administered in order to confer a high protection to the graft. During the post-transplant phase, Plasma Infusion prolonged the protection against complement activation. Calcineurin inhibitors (Tacrolimus 0.1mg/Kg/day) and Mycophenolic acid were introduced on the 4th post-transplant day. There was a fast steroid tapering, and the Thymoglobulin was maintained until the 4^th^ post-transplant day. A second Eculizumab (900mg) infusion was administered one week after the kidney transplantation, and then once a week for another two weeks, completing the attack scheme of 4 Eculizumab doses of 900 mg. Then the patient received Eculizumab 1200 mg every two weeks for another two months. After this time the Eculizumab administration was interrupted (August 2017). From August 2017 till now the patient has shown clinical stability, no disease recurrence, and a normal graft function.

**Figure S3 Complement Activation Products:** **A-C)** Complement C3a **(A)**, Ba **(B)** and sC5b-9 **(C)** fragments in serum of the patient (#2337 light gray boxes) collected three days after the second Eculizumab dose (at the 10^th^ post-transplant day), compared to NHS (white boxes). The C3a, Ba fragments in patient serum show values quite similar to NHS. Note that the sC5b-9 level in patient serum comes back to normal value under Eculizumab therapy. Results represent mean ±SD of three independent experiments.

## Supplementary Table 1

**Table 1. Clinical presentation and outcome**

| **Patient** | **#2337** |
| --- | --- |
| Sex | M |
| Age at onset (yr) | 22 |
| Initial Diagnosis | aHUS |
| Putative Trigger | infective |
| Onset Symptoms | face and legs swelling; purpuric rash; epistaxis; chest pain;  legs myalgia; postural instability |
| Serum Creatinine (mg /dl) | 16.85 |
| Hemoglobin (g/dl) | 7.2 |
| Platelets (10^3^ /µl) | 100 |
| LDH (U/L) | 1724 |
| Haptoglobin (g/L) | <0.08 |
| Schistocyte (%) | 10% |
| C3 (g/L) | 0.5 |
| Serum Calcium (mg/dl) | 8.86 |
| Serum Phosphate (mg/dl) | 4.8 |
| Nephrotic Syndrome | + |
| Acute Hemodialysis | + |
| PEX/PI | - |
| Immunosuppressive Drugs | - |
| Eculizumab | + |
| Dialysis | + |
| Time on Dialysis (months) | 23 |
| Renal Transplantation | 1 |
| CHM mismatches class1 | 1  (A11-B18) |
| CHM mismatches class2 | 0 |
| PRA before Transplantation | 0% |
| DSA | 0 |
| Recurrence | - |

M= male; aHUS= atypical Hemolytic Uremic Syndrome; sCr= creatinine; PEX=plasma exchange; PI= plasma infusion; PRA=panel reactive antibody; DSA= Donor-specific antibodies

+ indicates yes; and – indicates no.

**Table 2. Patient’s biological parameters during the 5 years of post-transplant follow-up**

|  | **26/05/2022** | **22/02/2021** | **23/10/2020** | **05/03/2019** | **05/02/2018** | **11/09/2017** | **20/07/2016** | **09/11/2015** | **08/06/2015** |
| --- | --- | --- | --- | --- | --- | --- | --- | --- | --- |
| **Hemoglobin (g/dl)** | 16.5 | 16.8 | 16.9 | 16.8 | 16.1 | 15.3 | 15.5 | 15.7 | 13.5 |
| **Platelets**  **(10^3 /µl)** | 226 | 241 | 225 | 196 | 195 | 221 | 226 | 219 | 190 |
| **LDH (UI)** | 201 | 179 | 198 | 200 | 187 | 203 | 186 | 199 | 212 |
| **Haptoglobin (g/L)** | 1.15 | 1.12 | 1.13 | 1.1 | 1.14 | 1.15 | 1.1 | 1.13 | 1.15 |
| **Schistocytes** | - | - | - | - | - | - | - | - | - |
| **C3 (g/l)** | 1.37 | 1.2 | 1.38 | 1.37 | 1 | 1.39 | 1.2 | 1.37 | 1.38 |
| **C4 (g/l)** | 0.32 | 0.34 | 0.36 | 0.32 | 0.4 | 0.33 | 0.32 | 0.32 | 0.34 |
| **Serum Creatinine (mg/dl)** | 1.29 | 1.12 | 1.16 | 1.08 | 1.37 | 1.19 | 1.14 | 1.29 | 1.3 |
| **Creatinine Clereance (ml/min)** | 127 | 139 | 111 | 108 | 115 | 140 | 109 | 112 | 96 |
| **Recurrence** | - | - | - | - | - | - | - | - | - |

+ indicates yes; and – indicates no.
